# Management of an Unusual Periprosthetic Giant Cell Tumor of Bone of the Proximal Tibia

**DOI:** 10.5435/JAAOSGlobal-D-18-00012

**Published:** 2018-09-12

**Authors:** Eric A. Chen, Dennis L. Caruana, Fazel A. Khan

**Affiliations:** From the State University of New York at Stony Brook University, Stony Brook, NY.

## Abstract

Giant cell tumor of bone is a relatively rare type of bone tumor, accounting for approximately 4.9% to 9% of all primary osseous neoplasms.^1^ Management options include intralesional curettage, or more uncommonly, wide resection. This process is then followed by reconstruction with bone graft or bone cementation. We present a case of giant cell tumor of bone adjacent to the tibial component of a preexisting total knee arthroplasty, treated with extensive curettage, argon beam coagulation, polymethyl methacrylate cementation with strut reinforcement, and mesh reconstruction of the extensor mechanism. Twenty months after treatment, the patient was recurrence free with a stable prosthesis and had return to functional activity. We report this treatment modality as a potentially effective method of approaching this rare orthopaedic entity.

Giant cell tumor of bone (GCTB) is a relatively rare type of bone tumor, accounting for approximately 4.9% to 9% of all primary osseous neoplasms, with an annual incidence in the United States of 1.6 per 10 million persons as determined by an analysis of the National Cancer Institute's Surveillance, Epidemiology and End Results Program. Incidence of GCTB is highest among adults aged 20 to 44 years (2.4 per 10 million per year) with a slightly higher rate of occurrence in women as compared with men but is also known to occur in older adults.^1,2^ GCTB is heterogeneous in histology and comprises three disparate cell lines: giant cell tumor stromal cells, multinucleated giant cells (MNGCs), and multinucleated histiocytic cells. The foremost cell line is the neoplastic component whereas the latter two are nonneoplastic. MNGCs—for which the name of this neoplasm is derived—might arise among the histological profile of other osseous neoplasms, as well as among cells which comprise nonneoplastic tissue. Therefore, diagnosis of a bone neoplasm as GCTB necessitates the neoplasm to be characterized by a ubiquity of MNGCs in a background consistent of giant cell tumor stromal cells and MNGCs.^3,4^

Although GCTB cases are usually benign and 90% of which develop at the epiphysis of the body's long bones, they tend to be aggressive and destructive in their expansion. GCTB cases occur with decreasing prevalence in the distal femur, proximal tibia, distal radius, and the sacrum; half of GCTB cases occur around the knee region. Only 16% of patients with GCTB, normally a localized neoplasm, are diagnosed with a distant metastatic process consistent with a primary GCTB, per surveillance, epidemiology and end result analysis, with pulmonary metastases as most common.^2,5,6^ We herein report a case whereby a total knee arthroplasty (TKA) was complicated by development of a GCTB necessitating reconstruction of the tibial component.

## Case Presentation

**Figure 1:**
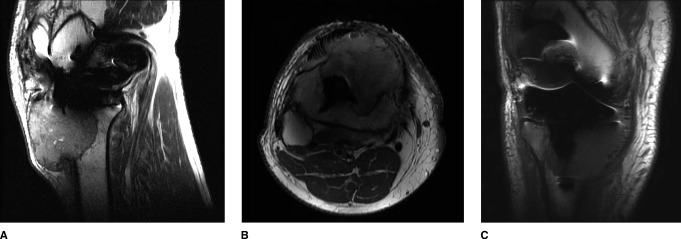
Preoperative MRI of the right knee. Sagittal Proton Dense (PD) (**A**), axial T1 Fat Supressed (FS) (**B**), and coronal T1 FS (**C**). Imaging demonstrates an erosive, well-circumscribed lesion extending from the infrapatellar fat pad into the proximal tibia metadiaphysis.

A 60-year-old man with a history of asthma, benign prosthetic hypertrophy, and hyperlipidemia presented 1.5 years after an uncomplicated primary right TKA done by an outside surgeon. He had been complaining of 4 months of increased pain in his right knee. An aspiration had been attempted, yielding 1 mL of sanguinous fluid which had not been sent for analysis. The patient continued to have swelling and increased pain in the knee, and an MRI was obtained demonstrating “pseudotumor” (Figure 1, A–C). He was then referred to our orthopaedic oncology office for further evaluation and management.

**Figure 2:**
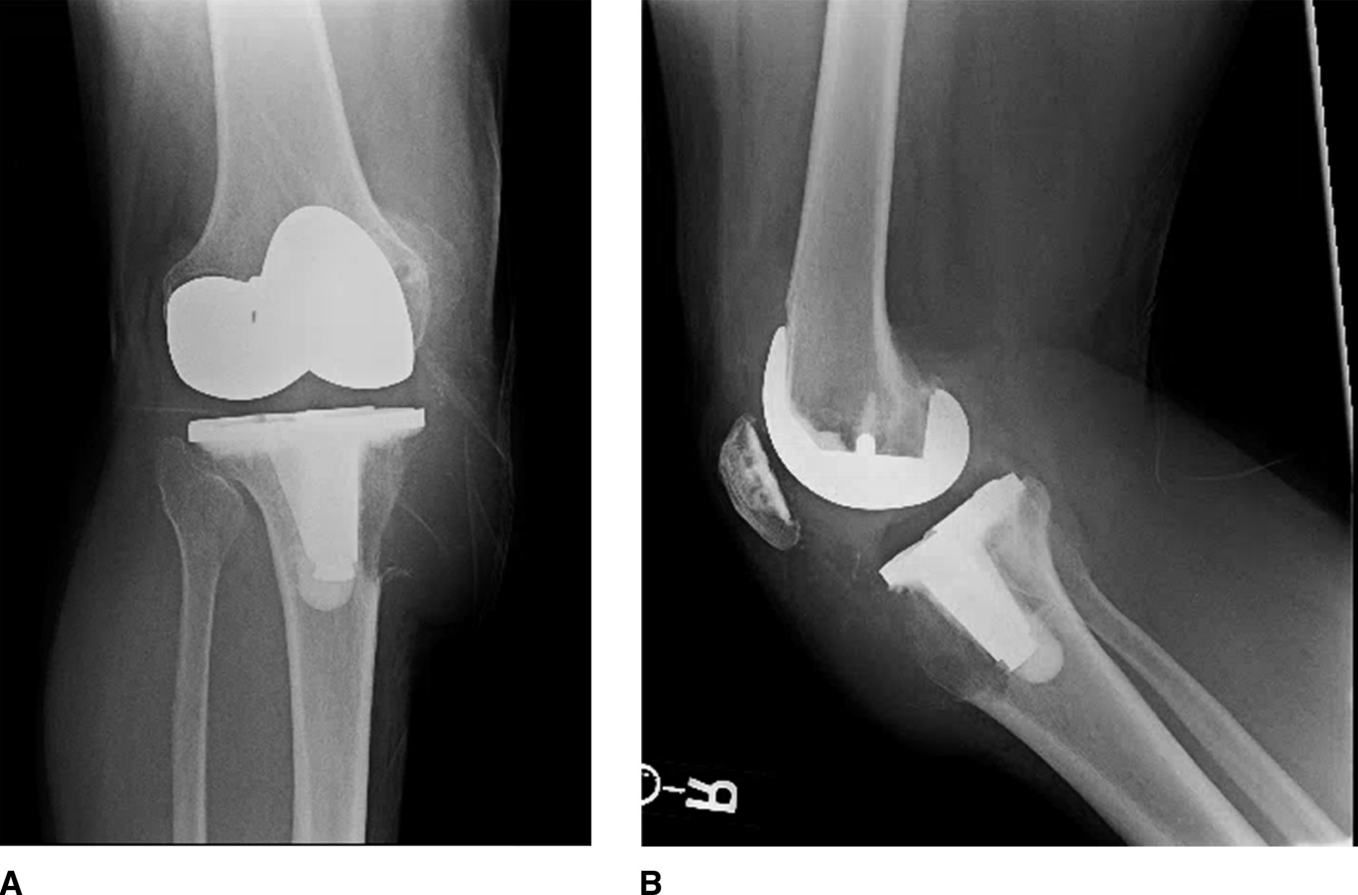
Preoperative radiographs of the right knee demonstrating a lytic juxtaprosthetic lesion of the proximal tibia.

**Figure 3:**
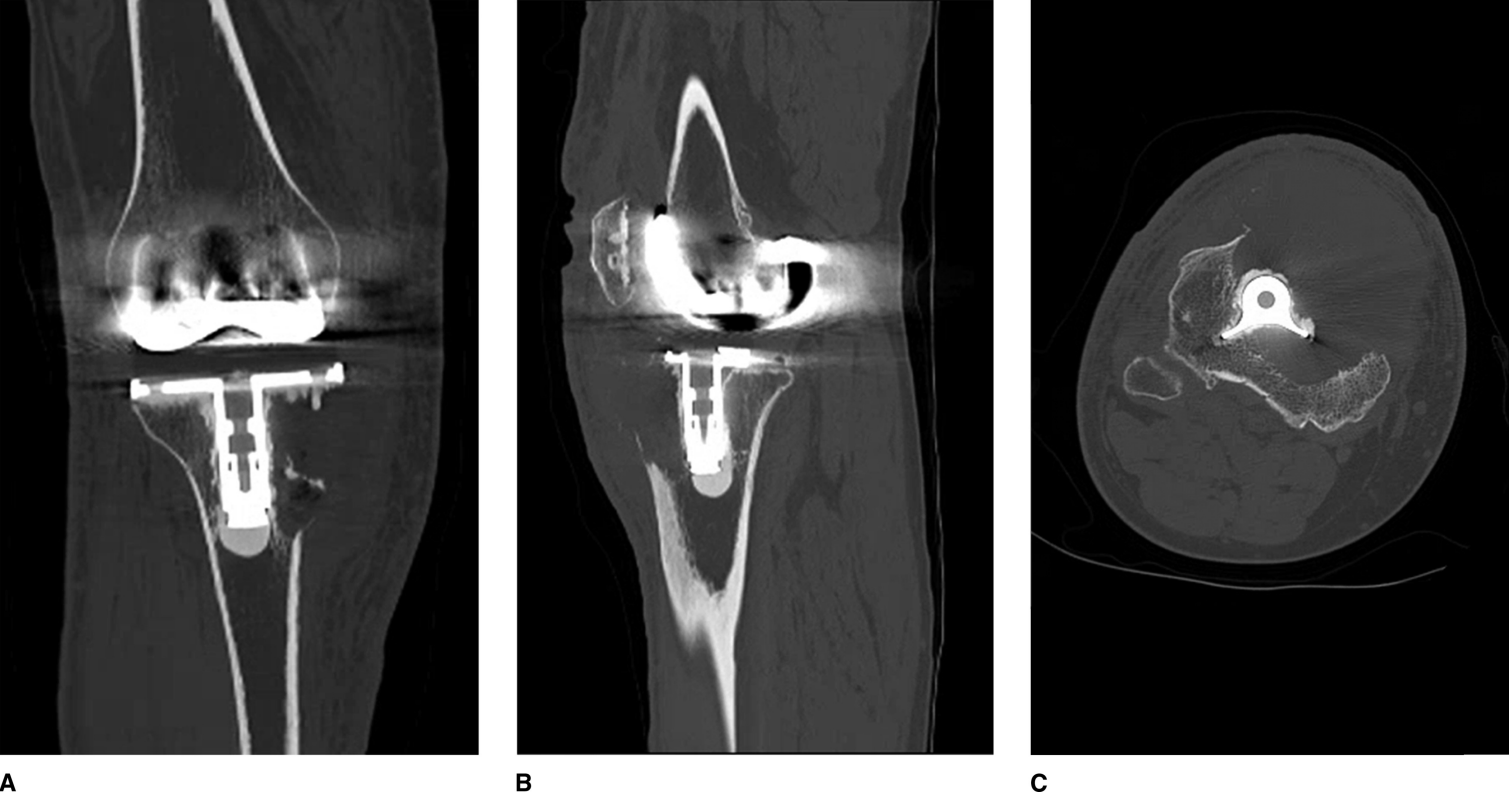
Coronal (**A**), sagittal (**B**), and axial (**C**) images of preoperative CT of the right knee demonstrating a large expansile destructive lytic lesion of the proximal tibia abutting the tibial component of the total knee arthroplasty.

**Figure 4:**
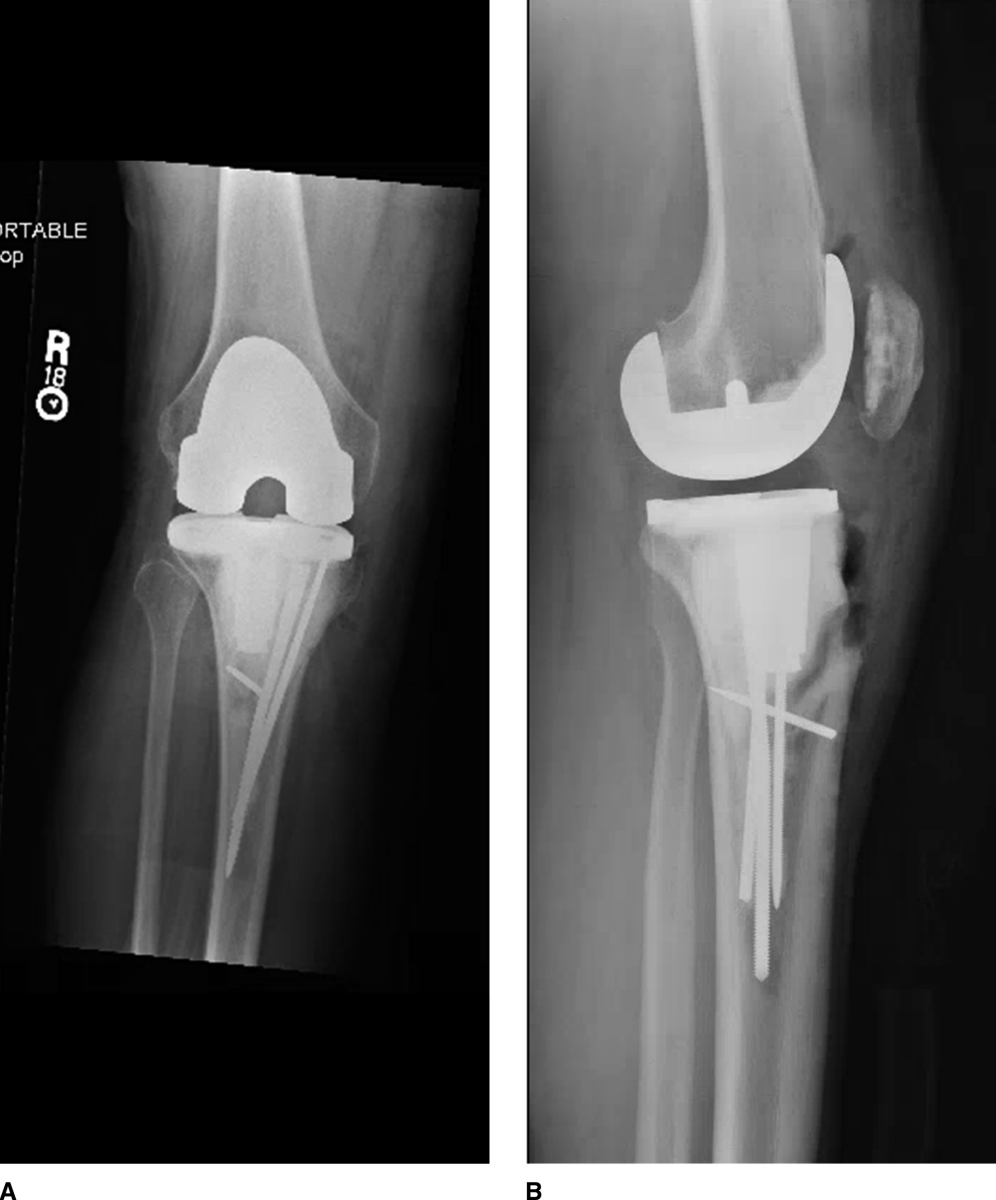
AP (**A**) and lateral (**B**) postoperative radiographs following revision of the tibial component with curettage, argon beam coagulation, cementation, Steinmann pin fixation, and extensor mechanism reconstruction.

After review of initial radiographs (Figure 2, A and B) and CT (Figure 3, A–C), the patient underwent an open biopsy of his right tibial lesion adjacent to the tibial baseplate one week after presentation to the office. Pathology from his initial biopsy was consistent with GCTB. One week following his open biopsy, the patient underwent a complex reconstruction of his proximal tibia as well as patellar tendon (Figure 4, A and B).

Intraoperatively, complete destruction of the medial cortex of the tibia was noted, with the area infiltrated extensively by tumor. After the initial anterior exposure through the previous TKA incision, the area was extensively curettaged. A high-speed burr and argon beam coagulator was then used to complete the resection at the edges of the cavity. Following the removal of the mass, we noted that the tibial baseplate was mechanically stable even after the extended curettage. An intraoperative determination was made to preserve the primary arthroplasty components and to reinforce the tibia with cement and Steinmann pin fixation. Steinmann pins were fired distally into the tibia, which allowed buttressing of the tibial baseplate proximally. The entire excisional cavity was then packed with polymethyl methacrylate (PMMA) cement. Intraoperative examination demonstrated that the construct had excellent stability and strength afterward.

Following reconstruction of the proximal tibia, attention was turned toward the patellar tendon. We noted that the destructive process had eroded much of the patellar tendon and reconstruction was required. Marlex mesh was used in the technique described by Browne and Hanssen.^7^ The mesh was layered into a construct with approximate width as the patellar tendon and then weaved into the remnant of the native patellar tendon into normal tendon tissue. #5 Ethibond suture was used to reinforce the closure and attachment of the Marlex mesh to the tendon, avoiding the placement of mesh adjacent to skin.

**Figure 5:**
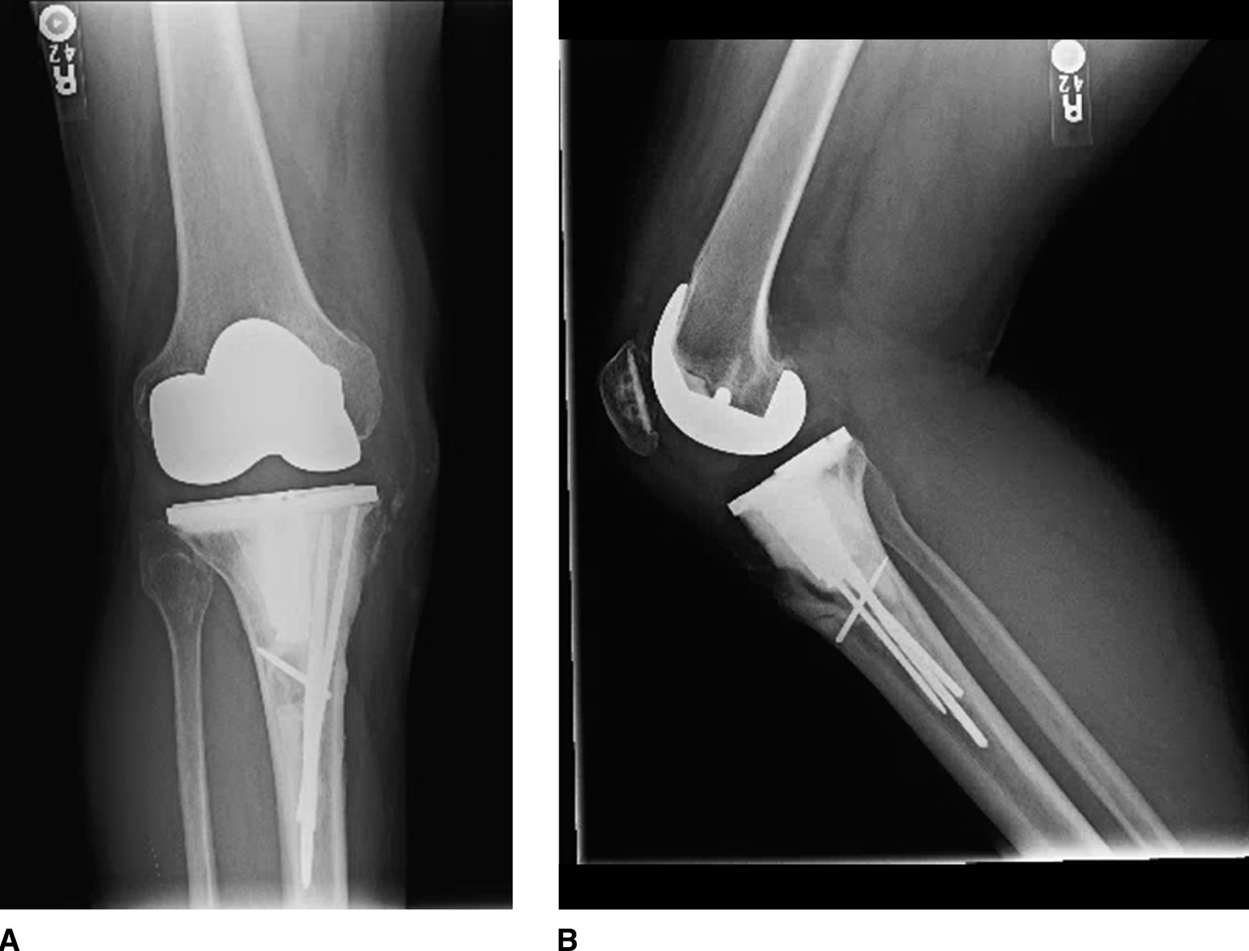
AP (**A**) and lateral (**B**) 16-month postoperative radiographs demonstrating maintained alignment without evidence of component subsidence or implant failure.

Before discharge, the patient was placed in a long leg bivalved cast. Three weeks postoperatively, the patient was transitioned into a hinged knee brace, which is locked in extension while upright. The patient was then instructed to allow for bed dangles with the knee. At 6 weeks post-op, the patient began physical therapy for gentle range of motion of the knee, still with brace locked in extension while ambulating. At 7 weeks, the patient was placed on Keflex for 1 week after he noticed a small amount of discharge from his distal incision site after a scab was removed, with resolution of symptoms. Three months post-op, the patient was allowed to weight bear as tolerated on his extremity. At this time, he was started on a trial of denusumab (Amgen Manufacturing Limited) adjuvant chemotherapy under the medial guidance of his oncologist. The patient developed a rash after two doses and was changed to zoledronic acid (Zometa; Novartis Pharmaceuticals Corporation) for a total of 6 months of diphosphonate therapy. He completed the course without further incident. Radiographs taken at 16 months demonstrated maintained alignment without evidence of component subsidence or implant failure (Figure 5, A and B). At a 20-month follow-up, the patient was weight bearing on the extremity without assistance, using a cane only for long distances.

## Discussion

Juxta-articular giant cell tumors of bone are a challenging entity in terms of its management. We report a case of a periprosthetic GCTB that arose in a proximal tibia adjacent to the cemented tibial component in a preexisting TKA. This orthopaedic entity has yet to be reported in the literature, and thus, no guidelines currently exist. The possibility of knee arthroplasty as a potential inciting factor for the development of malignancy is a concern that has been explored within the literature. Thus far, neither hip nor knee arthroplasty has been associated with the increased rate of development of malignancy over 10 years following arthroplasty.^8^

The treatment of a periprosthetic GCTB presents unique challenges in that reconstructive options are limited by both the location and healing capacity of such a lesion. Extensive curettage with adjuvant therapy and reconstruction is currently the standard of care for GCTB. However, with this treatment alone, recurrence rates can be as high as 60%.^9^ More recent studies have shown that a 5-year Kaplan-Meier disease-free survival estimate of 87.2% can be achieved with argon beam coagulation followed by PMMA cementation.^10^

In this case, reconstructive options were limited due to limited capability of ingrowth in the preexisting tibial baseplate design, as well as extensive destruction of the patellar tendon. Wide resection with complete removal of the tumor alongside the tibial baseplate followed by arthroplasty of the proximal tibia was considered during surgical planning. However, a decision was made preoperatively to pursue retention of the implant if it were found to be stable intraoperatively. Wide resection with revision of the tibial component would be reserved for future reconstructive attempts if necessary. This decision making was based on the possibility of increased risk of spread of the GCTB to the femoral side of the joint in the case that a conversion to a constrained prosthesis necessitating femoral sided revision was attempted. With extensive curettage with adjuvant argon beam coagulation followed by cementation of the tibial void with Steinmann pin reinforcement as well as mesh reconstruction of the patellar tendon, we were able to salvage and reconstruct the metaphyseal defect adjacent to the preexisting tibial implant. Postoperatively, the decision to restrict the patient’s weight-bearing status was made due to the need for maturation of the patient's reconstructed extensor mechanism.

## Conclusion

Periprosthetic giant cell tumors of bone are exceedingly rare and can pose unique challenges in terms of their management. In our case report, we found success in treating GCTB adjacent to the tibial component of a preexisting TKA with extensive curettage, argon beam coagulation, PMMA cementation with strut reinforcement, and mesh reconstruction of the extensor mechanism.
